# Role of Ser102 and Ser104 as Regulators of cGMP Hydrolysis by PDE5A

**DOI:** 10.1371/journal.pone.0107627

**Published:** 2014-09-23

**Authors:** Julie Carøe Nordgaard, Lars Schack Kruse, Steen Gammeltoft, Christina Rostrup Kruuse

**Affiliations:** 1 Lundbeck Foundation Center for Neurovascular signaling (LUCENS), Glostrup Research Institute, Glostrup Hospital, University of Copenhagen, Glostrup, Denmark; 2 Department of Clinical Experimental Research, Glostrup Hospital, University of Copenhagen, Glostrup, Denmark; 3 Department of Diagnostics, Clinical Biochemistry section Glostrup Hospital, University of Copenhagen, Glostrup, Denmark; 4 Department of Neurology, Neurovascular Research Unit, Herlev Hospital, University of Copenhagen, Herlev, Denmark; Center for Cancer Research, National Cancer Institute, United States of America

## Abstract

**Background:**

Phosphodiesterases (PDEs) cleave phosphodiester bonds in cyclic nucleotides and play diverse roles in cell biology. PDE5A is a cytoplasmic phosphodiesterase which specifically degrades cyclic guanosine monophosphate (cGMP), a cell signaling molecule that plays important roles in neuronal signaling and vascular smooth muscle contraction. Inhibition of PDE5A induces headache resembling migraine headaches.

**Aim:**

To test the hypothesis that Ser102 and Ser104 in PDE5A and/or their phosphoserine derivatives 1) regulate the intracellular localization and/or activity of PDE5A, and 2) modulate the interaction between PDE5A and pharmaceutical reagents in clinical or pre-clinical use for migraine headaches and other types of vascular dysfunction.

**Methods:**

Wild type PDE5A or PDE5A with substitution mutations (Ser102Ala, Ser104Ala or Ser102Ala/Ser104Ala) were overexpressed in SK-N-AS neuroblastoma cells as C-terminal fusions with green fluorescent protein. Transfected cells were treated with sildenafil, cilostazol, glyceryl trinitrate, calcitonin gene-related peptide (CGRP) or sumatriptan. PDE5A-GFP fusion proteins were localized in fixed cells by immunofluorescence and PDE activity was quantified in cell extracts by standard in vitro assay using [^3^H] cGMP.

**Results:**

The intracellular distribution of wild-type, single and double mutant PDE5A was similar and was not altered by exposure to sildenafil, cilostazol, glyceryl trinitrate, calcitonin gene-related peptide (CGRP) or sumatriptan. PDE5 activity was similar for wild type, Ser102Ala and Ser104Ala PDE5A, but activity of the Ser102Ala/Ser104Ala mutant was approximately two-fold higher than wild type. Double mutant Ser102Ala/Ser104Ala migrated as a single band on a native acrylamide gel, while wild-type and single mutant PDE5A migrated as a doublet.

**Interpretation:**

Ser102 and Ser104 may influence the conformational flexibility of PDE5A, which may in turn influence phosphorylation status, allosteric regulation by cGMP or other as yet unknown regulatory mechanisms for PDE5A. PDE5A activation could be important in reversal of migraine-like headache.

## Introduction

Headache and migraine-like pathophysiology can be induced in migraine patients by at least two endogenous signalling molecules, nitric oxide (NO) and calcitonin gene-related peptide (CGRP) [1]–[3]. CGRP initiates an intracellular signalling cascade predominantly involving cAMP [4], and NO increases intracellular levels of cGMP [5]. The levels of both cAMP and cGMP are modulated by phosphodiesterases (PDE). PDE5A is one of 11 PDEs identified to date, characterized by a ubiquitous cytosolic distribution and high specificity for cGMP. PDE5A is thought to have particular relevance to migraine headache pathophysiology and to possibly play a central role in the trigeminovascular system [6]–[8].

Sildenafil is a selective inhibitor of PDE5A used for treatment of erectile dysfunction and pulmonary hypertension [9]. Sildenafil induces headache in healthy subjects and migraine-like headache in migraine patients, without significant concomitant effect on cerebral artery hemodynamics [10]. The latter observation suggests that PDE5A and its downstream effects on cGMP- and NO-mediated signalling play critical roles in pain signalling during migraine-like attacks [8]; however, the exact details of its influence on pain signalling during headache and migraine events are not fully understood.

PDE5A is comprised of a C-terminal catalytic domain and an N-terminal domain containing two allosteric cGMP-binding sites, both involved in a cGMP negative-feedback loop [11].

PDE5A is phosphorylated at Ser102, by cGMP-stimulated protein kinase G (PKG) or cAMP-stimulated protein kinase A (PKA) [12], [13]. Although not shown to be phosphorylated *in vivo* or *in vitro* [13], Ser104 was predicted by bioinformatics analysis to be a high probability candidate phosphorylation site in PDE5A. The PDE5A protein sequence was run through the NetPhos 2.0 Server (Center for Biological Sequence Analysis, Danish Technical University) that predicts phosphorylation sites of proteins. PDE5A Ser104 gives a probability score of 0.992, while the confirmed site Ser102 gives a probability score of 0.996, thereby making Ser104 a likely candidate for phosphorylation. Biochemical studies have shown that binding of cGMP to the N-terminal allosteric sites and phosphorylation of Ser102 stimulate the activity of PDE5A [13], while the role of Ser104 remains unknown.

The goal of the present study was to investigate whether Ser102 and/or Ser104 in PDE5A, or their phosphorylated derivatives 1) regulate the intracellular localization and/or activity of PDE5A, and 2) modulate the interaction between PDE5A and sildenafil, cilostazol, glyceryl trinitrate, calcitonin gene-related peptide (CGRP) or sumatriptan, all of which are pharmaceutical reagents in clinical or pre-clinical use for migraine headaches and other types of vascular dysfunction. The results show no impact of single or double mutation of Ser102Ala and Ser104Ala on intracellular localisation of PDE5A or the response of the enzyme to the pharmaceutical agents tested in this study. However, the Ser102Ala/Ser104Ala double mutant was two-fold more active in hydrolysis of cGMP in cellular extracts than wild type or single mutant controls. The double Ser102Ala/Ser104Ala mutant migrated during native gel electrophoresis as a single band, whereas wild-type and single mutants migrated as two bands. The possible relevance of these results for understanding migraine headache-related cell signalling and management of migraine headache-related symptoms is discussed.

## Materials and Methods

### Cloning of PDE5A

An IMAGE clone for PDE5A was purchased from Geneservices Ltd. (clone no. 8991949, NCBI AC no. BC126233) and used as template for cloning and mutagenesis.

Primers for PDE5A amplification from IMAGE clone: forward 5′ NNNNGCTAGCATGGAGCGGGCCGG 3′ and reverse 5′ NNNNGGTACC GTTCCGCTTGGCCTGGC 3′. Expand High Fidelity DNA Polymerase (Roche,Hvidovre, Denmark) was used in a total volume of 50 µL for PCR. Annealing temperature was 53°C.

The PCR product was digested with NheI and KpnI and the resulting DNA fragment was inserted in-frame into the pGFP^2^ vector (BioSignal Packard, Skovlunde, Denmark) using quick ligation from New England Biolabs (Frankfurt am Main, Germany), such that the resulting proteins carried the PDE5A ORF followed by the GFP ORF in-frame. Ser102Ala, Ser104Ala and Ser102Ala/Ser104Ala mutations were introduced using the site-directed mutagenesis protocol from Stratagene (Agilent, Hørsholm, Denmark). After selection and purification, the construction of the recombinant clones was verified by DNA sequence analysis (Eurofins-MWG, Germany).

### Cell culture

The human neuronal cell line SK-N-AS, previously published in [14], [15], was kindly provided by Prof. Dr. Reinhard Wetzker (Institut für Molekulare Zellbiologie, Jena, Germany). Cells were cultivated in 50% IMDM and 50% Ham's F-12 media (Lonza, Vallensbaek strand, Denmark) containing 10% fetal bovine serum (Gibco, Taastrup, Denmark) and 25.000 U of a penicillin and streptomycin mixture (Lonza, Vallensbaek strand, Denmark) in a standard incubator (37°C, 5% CO_2_). The cells were split every seven days using trypsin-EDTA digestion.

### Transfection of SK-N-AS

SK-N-AS cells were seeded at a density of 100.000 cells/cm^2^. The following day the cells were transfected using 0.35 µg vector DNA and 1 µL PEI (Polyethyleneimine)/cm^2^. DNA and PEI were diluted separately in serum- and antibiotic- free medium (DMEM/F-12). The diluted DNA and PEI was mixed and vortexed. Following 15 min incubation at room temperature, the mixture was added to cells already in medium with FBS and without antibiotics. Transfected cells were incubated for 24 h prior to subsequent analyses.

### Visualization of Green Fluorescent Protein (GFP) tagged proteins

Transfected cells either were placed on ice. washed 3 times with ice-cold HBS (135 mM NaCl, 10 mM KCl, 0.4 mM MgCl_2_, 1 mM CaCl_2_, 10 mM Na-Hepes, pH 7.4) followed by 5 min at 4°C and 20 min at room temperature in 3.5 % paraformaldehyde. Cells were washed 3 times with HBS, incubated at −20°C for 6 min in 100% methanol, and washed once with HBS. Fixed cells were placed at 4°C, mounted on coverslips and examined using a fluorescence microscope.

### Activity assays

Cells were harvested in lysis buffer (150 mM NaCl, 50 mM Tris-HCl (pH 7.4), 0.5% Triton X-100) containing Complete protease inhibitor and PhosSTOP phosphatase inhibitor tablets (Roche, Hvidovre, Denmark) according to manufacturer's instruction and centrifuged at 14000 rpm for 20 min at 4°C. The lysates were kept on ice until the assay was started. PDE activity was quantified using the Phosphodiesterase [^3^H] cGMP SPA Enzyme Assay (GE Lifesciences, Skovlunde, Denmark) at room temperature for 15 min according to protocol. Radioactive [^3^H] was estimated using a MicrBeta2counter (Perkin Elmer, Skovlunde, Denmark).

### Denaturing gels, native gels and Western Blotting

Protein samples were adjusted to 25 µg/20 µL and separated using either a run using 4–12% SDS-PAGE gels (Kem-en-tec, Taastrup Denmark) run according to [16] or for native electroblotting NativePage Novex 3–12% Bis-Tris gels (Life Technologies, Nærum, Denmark) for 90 min at 40 mA and 180 V. Proteins were transferred to PVDF membranes by electroblotting for 1 h 10 min at 350 mA and 180 V. Membranes were blocked in ECL Advance blocking agent for 1 h prior to overnight incubation with primary antibody recognizing PDE5A at a dilution of 1∶300 (#PD5A-101AP, FabGennix, Frisco, USA) at 4°C on rotor. After a brief wash, the membranes were incubated with secondary HRP-conjugated donkey-anti-rabbit antibody (#N913V, GE Healthcare) at 1∶40.000 for 1 h at room temperature and developed using the ECL Advance detection kit (GE Lifesciences, Skovlunde, Denmark). Image capture was performed using a Fujifilm LAS-4000 (Fujifilm, A/S, Trørød, Denmark).

### Compounds

CGRP, DMSO and Cilostazol were from Sigma-Aldrich, Brøndby, Denmark. Gyceryl nitrate (GTN) was supplied by Amgros I/S, Copenhagen, Denmark. Sildenafil was a generous gift from Peter Sandner, Bayer HealthCare, Wuppertal. Sumatriptan was obtained from Glaxo Smith Kline, Brøndby, Denmark.

### Data and Statistics

Results are reported as mean ± SEM and were compared using Student's t-tests or one-way ANOVA. The differences were considered significant if *P*<0.05. Statistical tests were performed using GraphPad Prism software.

## Results

### Denaturing gel electrophoresis

**Figure 1:**
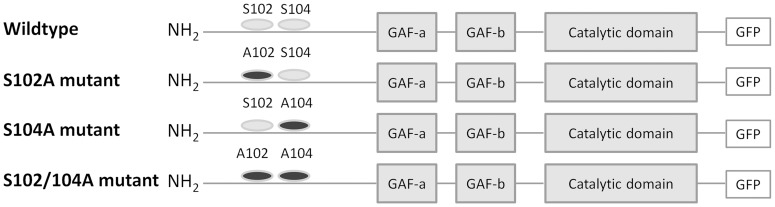
Schematic of PDE5A-GFP fusion proteins. A schematic representation of PDE5A-GFP fusion proteins used in this study. Ser102 and Ser104 are indicated by grey ovals or a black X, when substituted with alanine.

**Figure 2:**
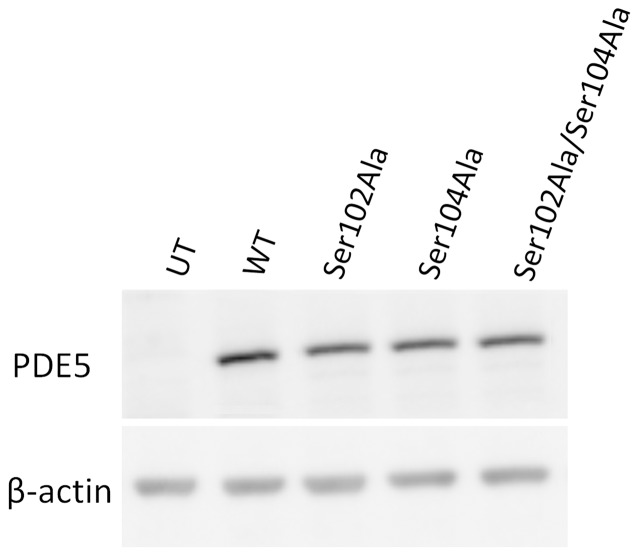
Denaturing gel electrophoresis of PDE5A-GFP fusion proteins in cell lysates. Denaturing gel electrophoresis (4–12%) and subsequent western blotting of cleared lysates. UT; Untransfected cell lysate, WT; wildtype PDE5A, Ser102A; Ser102A mutant PDE5A, Ser104A; Ser104A mutant PDE5A, Ser102/Ser104A; Ser102A/Ser104A mutant PDE5A.

SK-N-AS cells were transiently transfected with plasmids overexpressing wildtype, Ser102Ala, Ser104Ala or Ser102Ala/Ser104Ala PDE5A as GFP fusion proteins (Figure 1). Gel electrophoresis followed by Western blot analysis was performed on these cell lysates (Figure 2) to ensure that similar expression was seen for both wildtype and mutant proteins. When correcting for β-actin levels wildtype and mutant proteins were expressed in equal amounts.

### Localization of PDE5A in SK-N-AS cells

**Figure 3:**
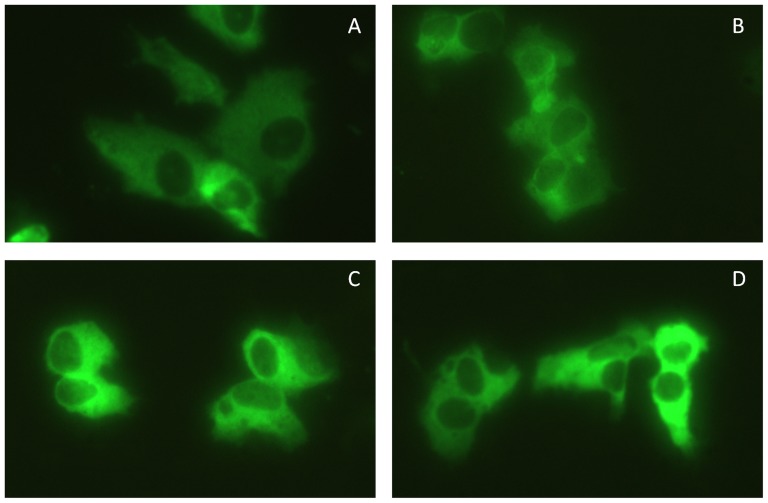
Localization of GFP-tagged PDE5A. SK-N-AS cells were transfected with constructs overexpressing PDE5A-GFP fusion proteins. **A**) wildtype PDE5A **B**) Ser102Ala PDE5A **C**) Ser104Ala PDE5A and **D**) Ser102Ala/Ser104Ala PDE5A. The images are at 40X magnification.

SK-N-AS cells were transiently transfected with plasmids overexpressing wildtype and mutant proteins. GFP fluorescence was visualized in fixed cells by fluorescence microscopy. The fluorescence data revealed similar cytoplasmic distribution for wild type and mutant PDE5A (Figure 3). Additional experiments showed that the subcellular distribution of wild type and mutant PDE5A did not change significantly when cells were cultured in the presence of sildenafil, GTN, CGRP, cilostazol or sumatriptan.

### cGMP hydrolysis and drug sensitivity of wild type and mutant PDE5A

**Figure 4:**
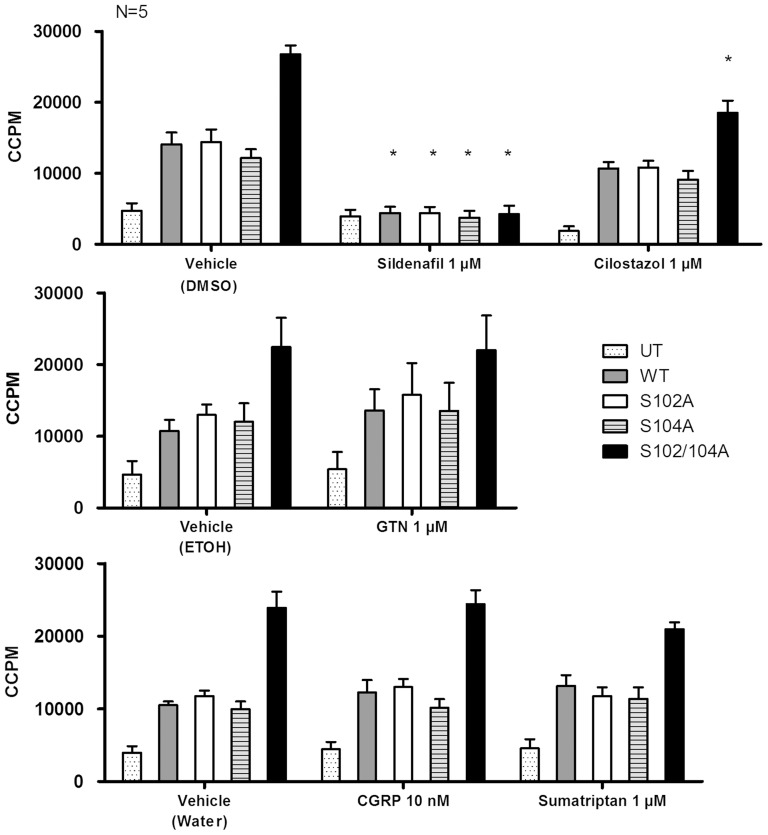
cGMP hydrolysis in the presence of substances related to migraine pathophysiology. Lysates from SK-N-AS neuronal cells were assayed for PDE activity using [^3^H] cGMP as substrate. PDE inhibitors were added as indicated. Vehicle controls were performed as indicated. Cell lysates were untransfected (UT) or overexpressed PDE5A or mutants as indicated. WT  =  Wildtype, S102A  =  Ser102Ala, S104A  =  Ser104Ala, S102/104A  =  Ser102Ala/Ser104Ala, ETOH  =  Ethanol, GTN  =  glycerylnitrate, CGRP  =  calcitonin gene-related peptide. The graphs show the mean ± SEM of five individual experiments. Significant differences from vehicle control are indicated by bar and asterisk (*).

Protein extracts were prepared from cells overexpressing wildtype and mutant PDE5A and the extracts were assayed for total cGMP hydrolysis activity in the absence and presence of sildenafil, GTN, CGRP, cilostazol or sumatriptan (Figure 4 and Table 1). Wildtype, single and double mutant PDE5A were significantly inhibited by 1 µM sildenafil (marked with an asterisk). In contrast,1 µM cilostazol, reported to inhibit PDE3 with IC_50_ = 0.2 nM and PDE5 with IC_50_ = 4.4 nM [17], under the conditions tested here, significantly inhibited only PDE5A Ser102Ala/Ser104Ala, with no significant effect on wild type, Ser102Ala or Ser104Ala PDE5A. For all other substances tested here, i.e. GTN (vehicle EtOH), CGRP (vehicle water) or sumatriptan (vehicle water), no significant impact on cGMP hydrolysis was observed. PDE5A Ser102Ala/Ser104Ala displayed a higher activity under all conditions, except in the presence of 1 µM sildenafil.

**Table 1:** cGMP hydrolysis by wild type PDE5A or PDE5A mutants in percent of control.

	Sildenafil	Cilostazol	GTN	CGRP	Sumatriptan
UT	27±7	75±11	−11±6	46±16	45±20
WT	69±4 (*)	51±15	−25±15	44±17	40±19
Ser102Ala	71±4 (*)	50±16	−12±17	46±16	52±14
Ser104Ala	69±5 (*)	53±14	−8±8	51±14	45±16
Ser102Ala/Ser104Ala	83±3 (*)	57±13 (*)	5±4	50±15	57±13

Data are shown as mean ± S.E.M and represent percentage inhibition of hydrolysis compared to control hydrolysis. All data are from five separate assays. Significant changes (p<0.05) compared to vehicle are marked with an asterisk (*). GTN; Glyceryl trinitrate, CGRP. Calcitonin gene-related peptide.

### Native gel electrophoresis

**Figure 5:**
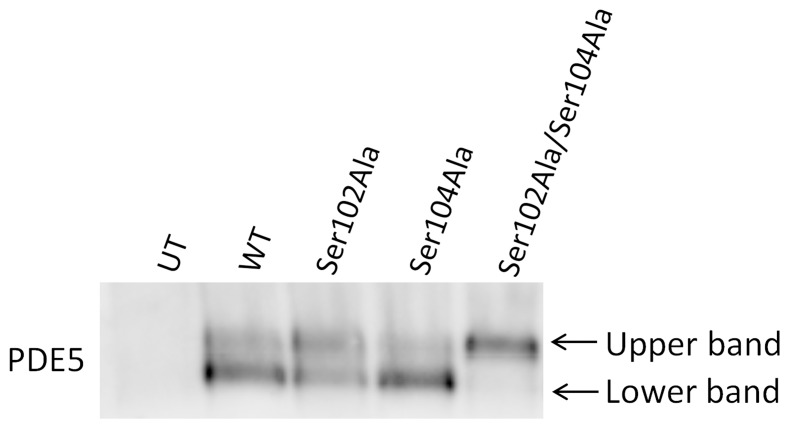
Native gel electrophoresis of PDE5A-GFP fusion proteins in cell lysates. Native gel electrophoresis (3–12% gel) and subsequent western blotting of cleared lysates. UT; Untransfected cell lysate, WT; wildtype PDE5A, Ser102Ala; Ser102Ala mutant PDE5A, Ser104Ala; Ser104Ala mutant PDE5A, Ser102Ala/Ser104Ala; Ser102Ala/Ser104Ala mutant PDE5A.

Native gel electrophoresis followed by Western blot analysis was performed on cell lysates containing wild type and mutant PDE5A-GFP fusion proteins (Figure 5). This experiment revealed that wild type, Ser102Ala and Ser104Ala PDE5A exist as both fast- and slow-migrating species during native gel electrophoresis, with greater abundance of the fast-migrating species for wildtype and Ser104Ala PDE5A. In contrast, Ser102Ala/Ser104Ala PDE5A exists as a single species, corresponding to the slower migrating form of the wild type enzyme. The cause of this mobility shift is not yet known (see discussion).

## Discussion

Phosphodiesterase 5A is one of the major cGMP hydrolyzing enzymes [18]. The aim of this study was to explore possible roles for Ser102 and Ser104 in regulating the activity or subcellular localization of PDE5A and/or the responsiveness of PDE5A to pharmaceutical agents that target cGMP or cAMP signaling cascades. The decision to explore the role of Ser102 was based on previous studies showing 1) that Ser102 is phosphorylated by PKA and PKG in vitro, and 2) that phosphorylation of Ser102 stimulates the activity of PDE5A [19]. Our bioinformatics analysis suggested that Ser104 also had a high probabiility as a PKA or PKG target (unpublished observations), leading us to investigate the impact of Ser102A, Ser104A or Ser102Ala/Ser104Ala double mutation on PDE5A functions, including susceptibility to substances related to migraine pathophysiology, subcellular localization, PDE activity and protein conformation.

Unexpectedly, in the present study, extracts from cells overexpressing PDE5A Ser102A demonstrate a similar level of cGMP hydrolysis activity as extracts overexpressing wild type PDE5A or Ser104A PDE5A. Though unverified, this may indicate that a relatively low fraction of total wild type PDE5A-GFP fusion protein is phosphorylated in extracts of SK-N-AS cells. Similarly, because Ser102Ala/Ser104Ala PDE5A had an unexpectedly high amount of PDE activity, mutation of Ser102 and Ser104 may allow the protein to adopt a conformation that support both higher activity. Conformational changes may lead to greater susceptibility to inhibition by other PDE inhibitors, such as seen with cilostazol (Figure 4). As wildtype and mutants are expressed in equal amounts (Figure 2), it appears that the increased activity may indeed be attributed the double deletion of serine and not simply explained by differences in expression levels.

Scaffolds that mediate intracellular signaling, including cross-talk between cAMP and cGMP signaling pathways, may be disrupted in cell lysates. One such scaffold is known as the calcitonin gene-related peptide receptor, a complex between calcitonin receptor-like receptor (CLR), receptor activity modifying protein 1 (RAMP1) and receptor component protein (RCP) [20]. CGRP signaling may also be disrupted, because activation of the CGRP receptor may not lead to activation of adenylate cyclase in cell extracts [21]. Secondly, over-expression of PDE5A relative to other signaling components of the cGMP and cAMP signaling pathways may alter normal patterns of signaling as well as cross-talk between signaling pathways.

Sumatriptan binds to subtypes of the serotonin receptor 5-HT_1D_ and 5HT_1B_. but the mechanism by which it exerts its downstream effects is still not known [22]. Therefore, it is not clear whether signaling events initiated by this agent in living cells would be conserved in cell extracts.

As noted above, cilostazol significantly inhibits cGMP degradation by Ser102Ala/Ser104Ala PDE5A, but does not inhibit the PDE activity of wildtype, Ser102Ala or Ser104Ala PDE5A. This supports the idea that mutating both Ser102 and Ser104 induces a conformational change that makes the active site of the double mutant more accesible to this compound than in wild type PDE5A, which binds cilostazol with an IC_50_ of 4.4 nM [17].

The wildtype and mutant cell lysates did not respond to GTN. Subsequent analysis revealed that soluble guanylate cyclase (sGC) is not present in SK-N-AS cells, which would explain this result, because it would limit cGMP hydrolysis in response to GTN (data not shown).

The strong inibition of Ser102Ala/Ser104Ala PDE5A by sildenafil may indicate that the Ser102Ala, Ser104Ala and Ser102Ala/Ser104Ala double mutations primarily affect the conformation of the N-terminal region of PDE5A, conserving the conformation of the catalytic domain and its susceptibility to sildenafil.

The native gel clearly showed marked difference in migration pattern for wildtype and mutants; the slower electrophoretic mobility of Ser102Ala/Ser104Ala PDE5A suggests greater positive surface charge or a more elongated conformation for wild type PDE5A than for the double mutant. The slower migrating species may also correspond to a more active state of PDE5A. Corbin et al. [23] reported that PDE5A adopts three distinct conformations, depending on exposure to metal ions, cGMP or sildenafil. They also propose that the band with the lowest mobility is the most active conformation of the enzyme.

**Figure 6:**
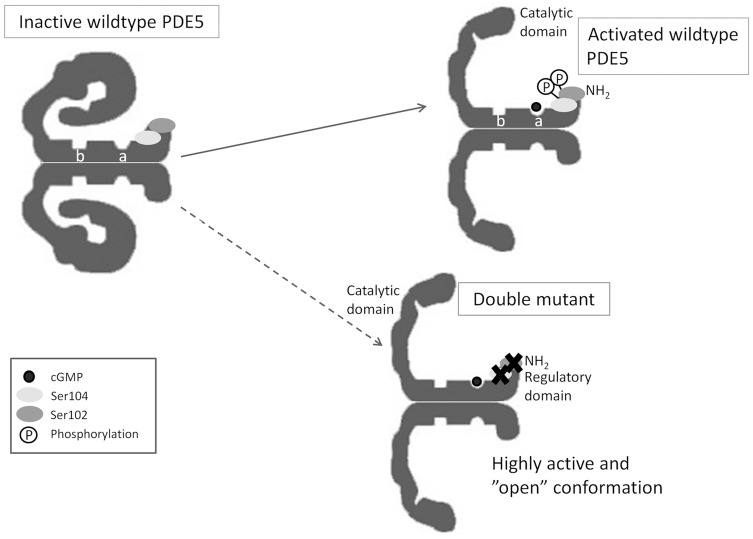
Proposed conformational model for PDE5A. PDE5A contains a C-terminal catalytic domain and an N-terminal domain containing two GAF (cGMP-binding PDE, Anabaena adenylyl cyclase, Escherichia coli FhlA protein) domains a and b. GAF a can bind cGMP. The binding site is involved in cGMP negative-feedback loop. PDE5A has a confirmed phosphorylation site at Ser102 and a putative site at Ser104. Mutating ser102 and ser104 leads to highly active protein possibly induced by conformational changes. Adapted from [23].

Thomas et al. [13] have previously shown that Ser104 is not phosphorylated *in vitro* by PKG, though they suggest that Ser104 could be phosphorylated by another kinase, such as PKA, in vivo. Ser104 may be of minor importance as a phosphorylation site, but instead playing a more important role in maintaining the tertiary structure of PDE5A. Under the conditions used in this paper, Ser102 and Ser104 appear equally important for regulation of PDE5A activity, and the Ser102Ala/Ser104Ala mutant is constitutively active (see figure 6 for proposed model). Further investigation could include activity measurements of knock-in mutants in which ser102 and ser104 is substituted by aspartic acid. Such would mimic phosphorylation of the residues, allowing for a better understanding of the significance of ser102 and ser104.

The current findings might lead to improved understanding of PDE5A catalysis and might support development of potent agents to activate specific PDEs. It also remains possible that compounds activating PDEs could have therapeutic applications, for example, in managing symptoms associated with migraine headaches [24], [25] or other conditions associated with high levels of cyclic nucleotides [26].
